# The impact of surgery on long-term survival of patients with primary intestinal non-Hodgkin lymphomas based on SEER database

**DOI:** 10.1038/s41598-021-02597-1

**Published:** 2021-11-29

**Authors:** Cuifen Zhang, Xiaohong Zhang, Zeyu Liu, Jiahao Tao, Lizhu Lin, Linzhu Zhai

**Affiliations:** 1 First Clinical Medical College, Guangzhou University of Chinese Medicine, Guangzhou, China; 2 Cancer Center, The First Affiliated Hospital of Guangzhou University of Chinese Medicine, Jichang Road 16#, Guangzhou, 510405 Guangdong China

**Keywords:** Cancer, Diseases, Medical research, Oncology

## Abstract

Evidence regarding the need for surgery for primary intestinal non-Hodgkin lymphoma (PINHL) patients with chemotherapy is limited and controversial. We aimed to investigate the specific impact of surgery on survival of PINHL patients. Data from PINHL patients (aged > 18 years) with chemotherapy between 1983 and 2015 were extracted from the Surveillance, Epidemiology, and End Results (SEER) database. We concerned about overall survival (OS) and improved cancer-specific survival (CSS). Propensity score matching (PSM) analysis was also used to explore the reliability of the results to further control for confounding factors. Finally, we screened 3537 patients. Multivariate regression analysis showed that patients with surgery and chemotherapy had better OS (hazard ratio [HR] 0.83; 95% confidence interval [CI] 0.75–0.93; p = 0.0009) and CSS (HR 0.87; 95% CI 0.77–0.99; p = 0.0404) compared with the non-operation group after adjusting for confounding factors. After PSM analysis, compared with non-surgery, surgery remained associated with improved OS (HR 0.77; 95% CI 0.68–0.87; p < 0.0001) and improved CSS (HR 0.82; 95% CI 0.72–0.95; p = 0.008) adjusted for baseline differences. In the large cohort of PINHL patients with chemotherapy older than 18 years, surgery was associated with significantly improved OS and CSS before and after PSM analysis.

## Introduction

Primary intestinal non-Hodgkin lymphoma (PINHL) is the most easily involved site of all extranodal lymphomas except the stomach. The incidence of extranodal lymphomas has been increasing among patients with non-Hodgkin lymphoma (NHL), reaching rates as high as 30–50%^1^. Among primary gastrointestinal non-Hodgkin lymphoma, 43–75% occur in the stomach, 10–35% in the small intestine, and 5–25% in the colon and rectum^2^. Intestinal lymphomas behave differently and have different survival rates compared with gastric lymphomas. Because of different anatomic locations in the gastrointestinal tract, PINHL mainly comprises diffuse large B cell lymphoma (DLBCL) and T cell lymphoma, while gastric lymphomas are mostly represented by the mucosa-associated lymphoid tissue (MALT) subtype. Also, gastric lymphoma doesn’t show better outcomes with chemotherapy and surgery which may be different from PINHL^3,4^. Therefore, it is particularly significant to conduct a study targeting PINHL as a separate entity^5^.

However, considering that very few, small, and single-center studies were conducted to access the optimal treatment practices in PINHL patients, there is lack of sufficient and strong evidence to guide clinical diagnosis and treatment^5^. For example, several studies supported that surgery combined with chemotherapy could improve overall survival^1,6–8^; however, others showed no impact on patients’ survival^9–11^. Several studies suggested that only some patients, who had a localized or early-stage disease, could gain better prognosis^2,12–14^. In summary, selection of appropriate therapeutic regimens to improve survival in PINHL patients is still undefined. Here, we retrospectively assessed the impact of surgical and non-surgical management on survival outcomes in a large cohort of PINHL patients who had received chemotherapy in the Surveillance, Epidemiology, and End Results (SEER) Program.

## Results

### Characteristics of patients and disease

**Figure 1:**
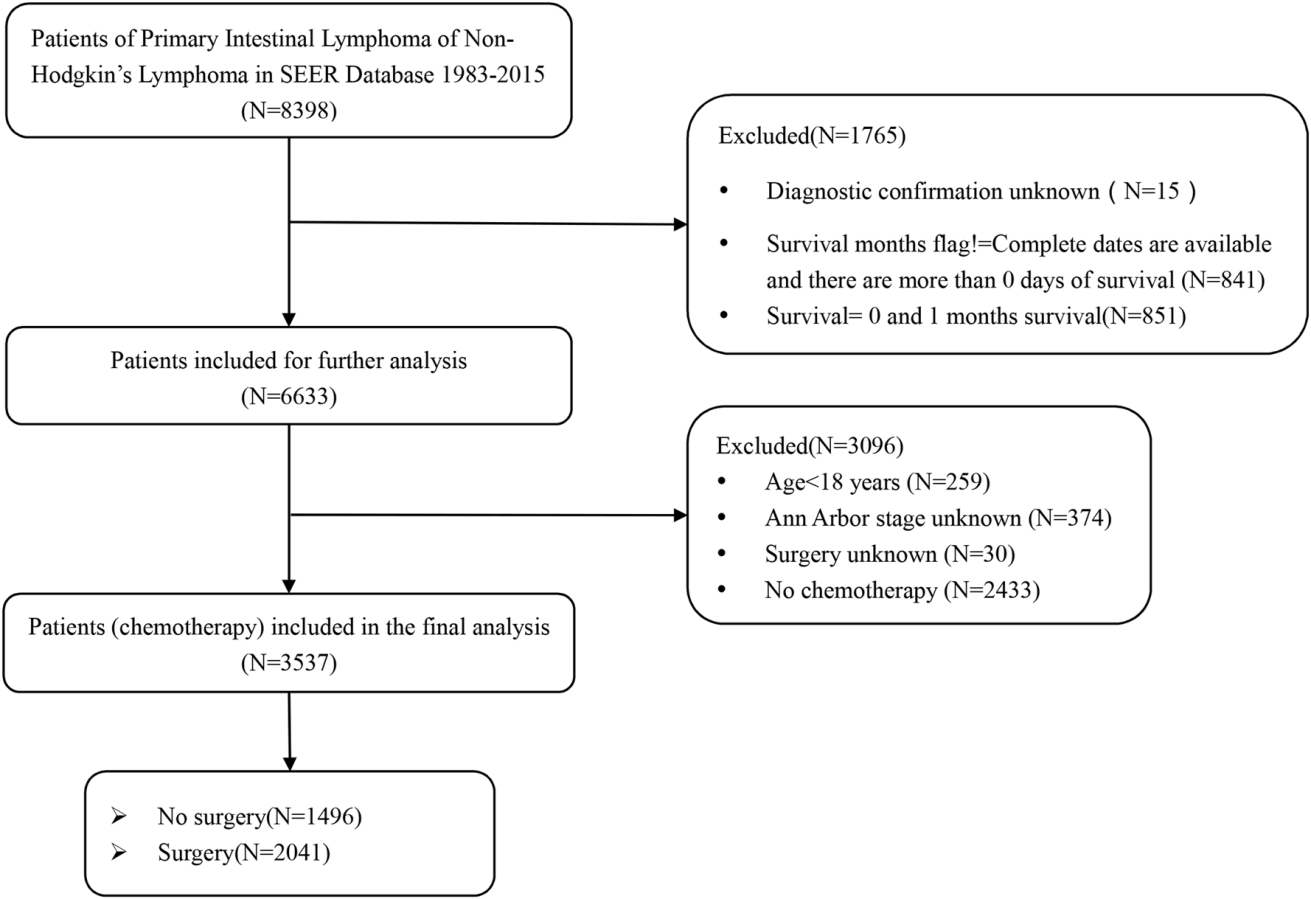
The Flowchart of inclusion and exclusion criteria.

A total of 3537 patients were extracted in the final analysis (Fig. 1). The characteristics of patients are listed in Table 1. There were 57.70% (n = 2041) of patients who had received surgery, while 1496 patients had not undergone surgery. The mean age was 58.87 years. Male patients were more frequent than females, accounting for 65.51% (n = 2317). More than 80% of patients (81.88%, n = 2896) were White. There were 2061 married patients (58.27%). As time went by, the combination of chemotherapy and surgery was accepted by more patients rather than chemotherapy alone. Specifically, 1.80% of patients (n = 27) were treated by chemotherapy alone, while 5.39% of patients (n = 110) were given treatment including surgery and chemotherapy in the 1980s. In 2000s, 719 patients accepted chemotherapeutic treatment, and 998 patients underwent surgery plus chemotherapy. There were 34.72% (n = 1228) patients in Ann Arbor stage I and 31.78% (n = 1124) in stage II, which revealed that diseases were mainly diagnosed at an early stage. In total, 1873 patients (52.95%) were diagnosed with diffuse large B-cell lymphoma. The disease location in the majority of patients (57.22%; n = 2024) was in the small intestine. Only 8.06% (n = 285) received radiation plus chemotherapy. Overall, both groups were balanced concerning patient demographic characteristics. However, small but statistically significant inter-group differences were observed in tumor characteristics and treatment modality.

**Table 1:** Baseline patient demographic and clinical characteristics.

	Total (n = 3537): n (%)	Non-surgery (n = 1496): n (%)	Surgery (n = 2041): n (%)	p-value
Age (years)	58.87 ± 16.00	58.91 ± 15.97	58.84 ± 16.02	0.899
**Gender**				0.086
Female	1220 (34.49%)	492 (32.89%)	728 (35.67%)	
Male	2317 (65.51%)	1004 (67.11%)	1313 (64.33%)	
**Race**				0.365
White	2896 (81.88%)	1209 (80.82%)	1687 (82.66%)	
Black	220 (6.22%)	105 (7.02%)	115 (5.63%)	
Other	403 (11.39%)	174 (11.63%)	229 (11.22%)	
Unknown	18 (0.51%)	8 (0.53%)	10 (0.49%)	
**Marital status**				0.326
Unmarried	1361 (38.48%)	597 (39.91%)	764 (37.43%)	
Married	2061 (58.27%)	852 (56.95%)	1209 (59.24%)	
Unknown	115 (3.25%)	47 (3.14%)	68 (3.33%)	
**Year of diagnosis**				< 0.001
1980s	535 (15.13%) 137 (3.87%)	27 (1.80%)	110 (5.39%)	
1990s		198 (13.24%)	337 (16.51%)	
2000s	1717 (48.54%)	719 (48.06%)	998 (48.90%)	
2010s	1148 (32.46%)	552 (36.90%)	596 (29.20%)	
**Ann Arbor stage**				< 0.001
I	1228 (34.72%)	554 (37.03%)	674 (33.02%)	
II	1124 (31.78%)	381 (25.47%)	743 (36.40%)	
III	246 (6.96%)	105 (7.02%)	141 (6.91%)	
IV	939 (26.55%)	456 (30.48%)	483 (23.66%)	
**Histologic**				< 0.001
DLBCL	1873 (52.95%)	720 (48.13%)	1153 (56.49%)	
FL	426 (12.04%)	209 (13.97%)	217 (10.63%)	
MCL	200 (5.65%)	134 (8.96%)	66 (3.23%)	
BL	228 (6.45%)	69 (4.61%)	159 (7.79%)	
TCL	170 (4.81%)	47 (3.14%)	123 (6.03%)	
Other	640 (18.09%)	317 (21.19%)	323 (15.83%)	
**Tumor site**				< 0.001
Small bowel	2024 (57.22%)	775 (51.80%)	1249 (61.20%)	
Ileocecum	592 (16.74%)	179 (11.97%)	413 (20.24%)	
Colon	648 (18.32%)	338 (22.59%)	310 (15.19%)	
Other	273 (7.72%)	204 (13.64%)	69 (3.38%)	
**Radiation**				< 0.001
No	3252 (91.94%)	1324 (88.50%)	1928 (94.46%)	
Yes	285 (8.06%)	172 (11.50%)	113 (5.54%)	

*DLBCL* diffuse large B cell, *FL* follicular lymphoma, *MCL* Mantle cell lymphoma, *BL* Burkitt lymphoma, *TCL* T cell lymphoma.

Univariate analysis identified that disease diagnosed from 1983 to 2015, younger age, female sex, Caucasian, married status, and the early stage of disease were associated with better survival in OS and CSS, and use of radiation was associated with poor CSS (Table 2).

**Table 2:** Univariate analysis of OS and CSS Cox proportion hazard ratio analysis before propensity score matching.

Characteristics	OS		CSS	
	HR (95% CI)	p value	HR (95% CI)	p value
Age(years)	1.03 (1.02, 1.03)	< 0.0001	1.01 (1.01, 1.02)	< 0.0001
**Gender**				
Female	1		1	
Male	1.20 (1.08, 1.33)	0.0009	1.33 (1.17, 1.51)	< 0.0001
**Race**				
White	1		1	
Black	1.28 (1.06, 1.54)	0.0116	1.32 (1.06, 1.64)	0.0135
Other	0.88 (0.75, 1.03)	0.1138	0.97 (0.81, 1.17)	0.7463
Unknown	0.41 (0.13, 1.27)	0.1223	0.55 (0.18, 1.72)	0.3085
**Marital status**				
Unmarried	1		1	
Married	0.80 (0.72, 0.89)	< 0.0001	0.73 (0.64, 0.82)	< 0.0001
Unknown	0.79 (0.58, 1.06)	0.1204	0.75 (0.53, 1.06)	0.105
**Year of diagnosis**				
1980s	1		1	
1990s	1.04 (0.84, 1.28)	0.7349	1.22 (0.93, 1.58)	0.1449
2000s	0.53 (0.43, 0.65)	< 0.0001	0.56 (0.44, 0.73)	< 0.0001
2010s	0.37 (0.30, 0.47)	< 0.0001	0.37 (0.28, 0.48)	< 0.0001
**Ann Arbor stage**				
I	1		1	
II	1.02 (0.90, 1.16)	0.7299	1.13 (0.97, 1.31)	0.1222
III	1.03 (0.92, 1.40)	0.2524	1.31 (1.03, 1.67)	0.0293
IV	1.62 (1.43, 1.83)	< 0.0001	1.81 (1.57, 2.10)	< 0.0001
**Histologic**				
DLBCL	1		1	
FL	0.42 (0.34, 0.52)	< 0.0001	0.30 (0.24, 0.43)	< 0.0001
MCL	0.89 (0.72, 1.12)	0.3207	0.95 (0.74, 1.23)	0.7009
BL	0.77 (0.61, 0.96)	0.0229	0.84 (0.65, 1.09)	0.2014
TCL	2.52 (2.09, 3.03)	< 0.0001	2.93 (2.40, 3.59)	< 0.0001
Other	1.19 (1.05, 1.35)	0.0062	1.25 (1.08, 1.46)	0.0028
**Tumor site**				
Small bowel	1		1	
Ileocecum	0.90 (0.79, 1.04)	0.1623	0.80 (0.67, 0.95)	0.01
Colon	1.20 (1.05, 1.36)	0.0067	1.11 (0.95, 1.29)	0.1984
Other	1.37 (1.16, 1.64)	0.0003	1.48 (1.22, 1.80)	< 0.0001
**Radiation**				
No	1		1	
Yes	1.11 (0.94, 1.32)	0.2277	1.25 (1.03, 1.52)	0.0263

*OS* overall survival, *CSS* cancer specific survival, *HR* hazard ratio, *DLBCL* diffuse large B cell lymphoma, *FL* Follicular lymphoma, *MCL* Mantle cell lymphoma, *BL* Burkitt lymphoma, *TCL* T cell lymphoma.

### Primary outcomes

Adjusting for the corresponding variables that affected the survival in the surgically treated group, multivariate regression analysis showed that the surgery group was associated with an improved OS (HR 0.83; 95% CI 0.75–0.93; p = 0.0009) and a trend toward improved CSS (HR 0.87; 95% CI 0.77–0.99; p = 0.0404) compared with the non-operation group (Table 3). Moreover, surgery was associated with significantly lower risk of overall (HR 0.77; 95% CI 0.68–0.87; p < 0.0001) and cancer-specific death (HR 0.82; 95% CI 0.72–0.95; p = 0.008) adjusted for baseline differences compared with non-surgery users in the PSM cohort (Table 4).

**Table 3:** Multivariable Analysis of OS and CSS in Non-surgery group and surgery group.

Treatment^a^	Non-adjusted	p-value	Adjust I^a^	p-value	Adjust II^b^	p-value
**OS**						
Non-surgery	1		1		1	
Surgery	0.92 (0.84, 1.02)	0.1227	0.94 (0.85, 1.03)	0.1951	0.83 (0.75, 0.93)	0.0009
**CSS**						
Non-surgery	1		1		1	
Surgery	0.96 (0.85, 1.08)	0.4634	0.98 (0.87, 1.10)	0.6767	0.87 (0.77, 0.99)	0.0404

*OS* overall survival, *CSS* cancer specific survival.

^a^There were 2041 and 1496 patients in unmatched surgery and non-surgery groups, respectively.

^b^Adjust I model of P values adjusted for age, gender, race and marital status.

^c^Adjust II model of P values adjusted for age, gender, race, marital status, year of diagnosis, Ann Arbor Stage, histologic, tumor site and radiation.

**Table4:** Multivariable Analysis of OS and CSS in Non-surgery group and surgery group after matching.

Treatment^a^	Non-adjusted	p-value	Adjust	p-value
**OS**				
Non-surgery	1		1	
Surgery	0.94 (0.84, 1.05)	0.2497	0.77 (0.68, 0.87)^b^	< 0.0001
**CSS**				
Non-surgery	1		1	
Surgery	0.99 (0.87, 1.13)	0.9221	0.82 (0.72, 0.95)^c^	0.008

*OS* overall survival, *CSS* cancer specific survival.

^a^After PSM with 1:1 ratio, there were both 1434 patients in the matched surgery and non-surgery groups, respectively.

^b^Adjusted for age, year of diagnosis, Ann Arbor Stage, histologic and tumor site.

^c^Adjusted for age, gender, marital status, year of diagnosis, Ann Arbor Stage, histologic, tumor site and radiation.

**Figure 2:**
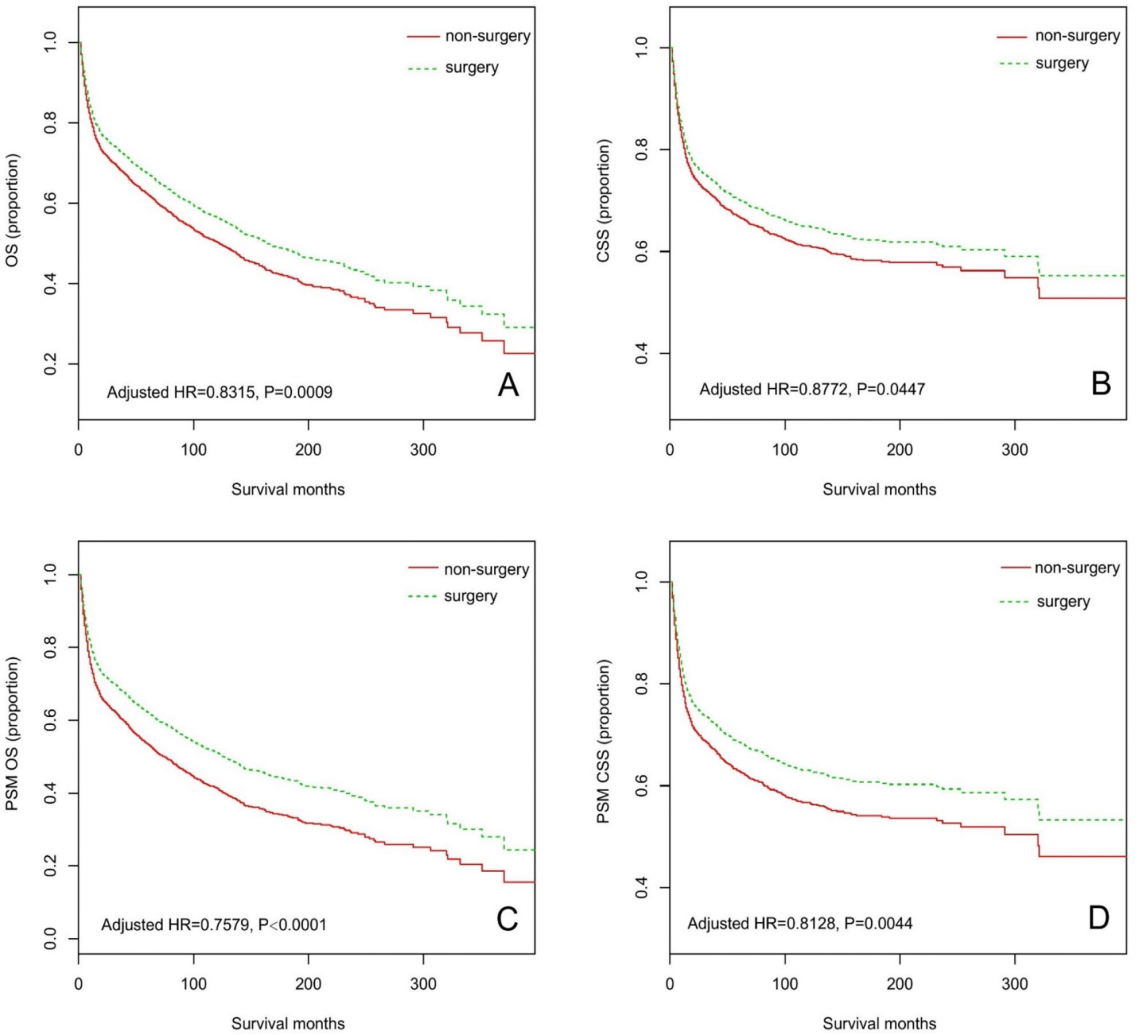
Kaplan–Meier curves for (**A**) overall survival (OS) and (**B**) cancer specific survival (CSS), for (**C**) propensity score-matching (PSM) OS and (**D**) PSM CSS. Adjusted HR was calculated based on the Cox proportional hazard model with adjustment of age, gender, race, marital status, year of diagnosis, Ann Arbor stage, histologic, tumor site and radiation in Figure (**A,B**). Figure (**C**) of OS adjusting for age, year of diagnosis, Ann Arbor stage, histologic and tumor site. Figure (**D**) of CSS adjusting for age, gender, marital status, year of diagnosis, Ann Arbor stage, histologic, tumor site and radiation.

Median survival for the entire cohort was 53 months (4.4 years; range, 2–397 months). Kaplan–Meier survival curve after adjusting for confounders demonstrated a statistically significant improvement in OS by surgery, with a median OS of 123 months in the non-surgery group vs. 163 months in the surgery group (HR 0.8315; 95% CI 0.7460–0.9268; p < 0.0009; Fig. 2A). We also observed a significant improvement in CSS with the use of surgery (HR 0.8772; 95% CI 0.7719–0.9969; p = 0.0447; Fig. 2B).

### Stratified analysis

**Figure 3:**
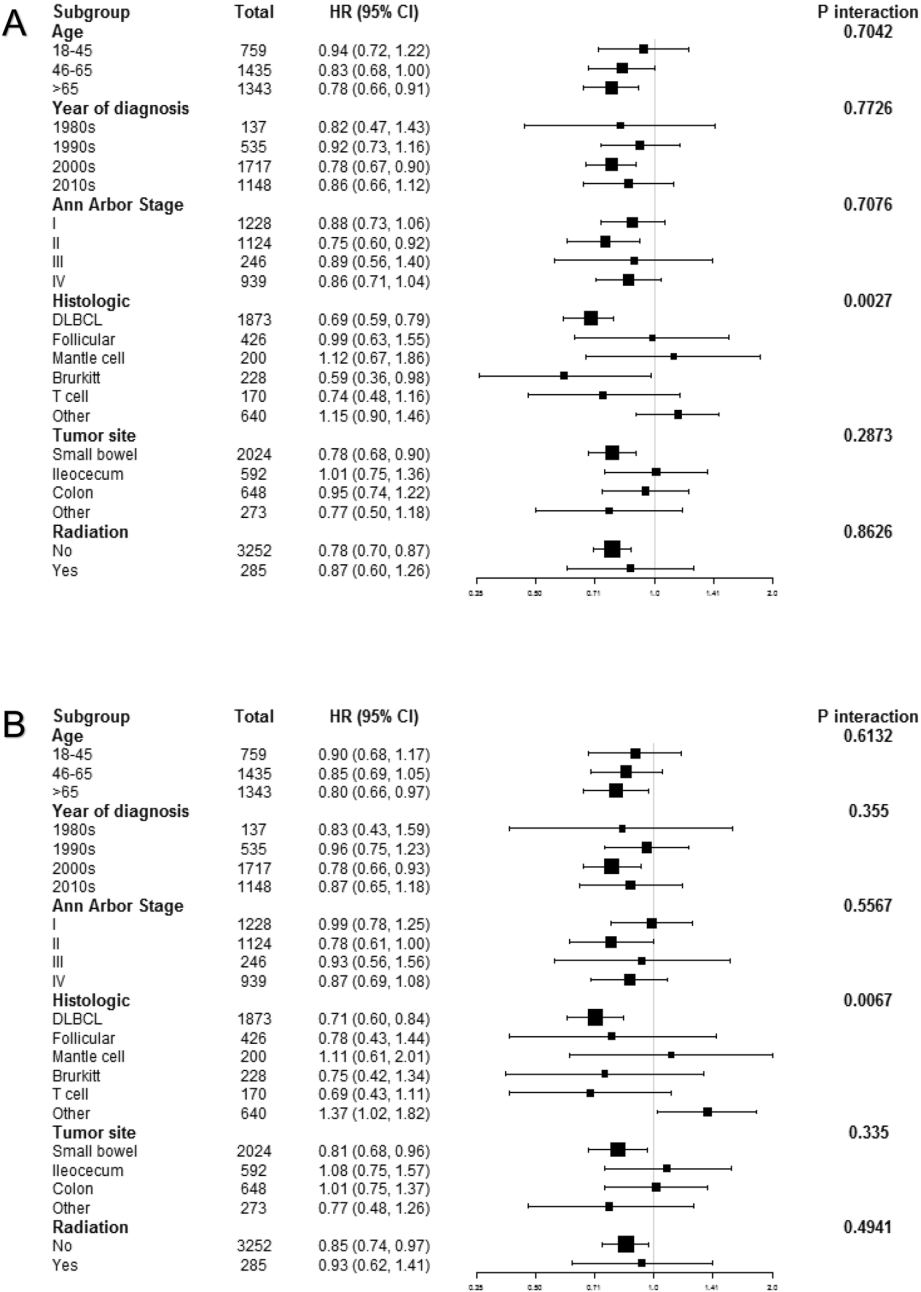
Forest plots for (**A**) overall survival (OS) and (**B**) cancer specific survival (CSS) with surgery plus chemotherapy treatment. Adjusted HR was calculated based on the Cox proportional hazard model with adjustment of age, gender, race, marital status, year of diagnosis, Ann Arbor stage, histologic, tumor site and radiation except the subgroup variable in Figure (**A,B**).

When the analysis was restricted to the subgroups displayed in the form of a forest plot, the HR for OS and CSS did not change significantly, and all the subgroups demonstrated improved OS and CSS with surgery. Surgery was steadily associated with better survival in the subgroup analyses based on various variables with P for interaction > 0.05, except for histology. We accessed the effect of varying HRs and differences in the rate of histology on the estimated OS and CSS effect of surgery. We were surprised to find out that patients with diffuse large B cell lymphoma could significantly benefit from surgery combined with chemotherapy, considering the improved OS (HR 0.69; 95% CI 0.59–0.79; p for interaction = 0.0027) and CSS (HR 0.71; 95% CI 0.60–0.84; p for interaction = 0.0067) compared with the non-surgery group (Fig. 3).

### Survival after PSM analysis

The PSM model was operated to minimize the differences in baseline characteristics and validate the outcome reliability. A total of 2041 patients who received surgical treatment could be matched to comparator patients, who did not receive surgery, by using a matching criterion of ± 0.05 of the propensity score. In the groups matched for the propensity score, 1434 patients who underwent surgery were matched with 1434 patients who did not. After matching, the standardized differences were less than 10.0% for several variables, while other might still display differences between the two groups (Supplemental Table S1). To further verify the results, a variety of analyses were carried out after adjusting for the variables.

In the PSM cohort, univariate survival analysis indicated that younger age, female sex, White patients, married individuals, and the early stage were important prognostic factors for OS and CSS (Supplemental Table S2). After PSM analysis (n = 2868), median OS improved from a median of 75 months in the non-surgery group to 126 months in the surgery group (HR 0.7579; 95% CI 0.6712 to 0.8560, p < 0.0001; Fig. 2C). Improvement in CSS was also observed (HR 0.8128; 95% CI 0.7049 to 0.9373; p = 0.0044; Fig. 2D).

The results of stratification in OS and CSS after adjusting for confounders in the PSM cohort are displayed in the forest plot in Supplemental Figure**.** Surgery plus chemotherapy also led to better OS and CSS than chemotherapy alone through in different stratification. Similar to Fig. 3, after adjusting for other potential confounders, patients with diffuse large B cell lymphoma could have better OS (HR 0.66; 95% CI 0.56 to 0.77; p for interaction = 0.0018) and CSS (HR 0.68; 95% CI 0.56 to 0.82; p for interaction = 0.0019) by undergoing surgery plus chemotherapy treatment compared with chemotherapy alone. The effect of surgery on OS and CSS was the same as a result before the matching.

## Discussion

Primary gastrointestinal lymphoma is known as the most common extranodal NHL. Intestinal lymphoma appears to be rare compared with gastric lymphoma. Primary intestinal lymphoma has often been presented as a subgroup of gastrointestinal lymphoma in the study of primary gastrointestinal lymphoma, which mainly reported the optimal treatment algorithms aimed at gastric lymphoma; in contrast, studies of primary intestinal lymphoma alone are absent^5^. Besides, substantial progress has been achieved by the use of various conservative treatments over time^15,16^, which means that surgical treatment for the survival of PINHL may require transvaluation. At present, due to the effect of anti HP treatment and the application of rituximab, retaining stomach function was possible and the role of surgery should be reevaluated. The results^17^ showed that only 8% of non-surgical patients had serious gastrointestinal complications after chemotherapy and underwent emergency surgery, while 57.78% of surgical patients appeared varying degrees of gastrointestinal complications, indicating that chemotherapy had obvious advantages in safety and long-term quality of life for gastric lymphoma patients^18^. DLBCL is the most common NHLs, which has often been studied. Considering different clinical characteristics of gastric and intestinal lymphoma, the treatment strategy for primary gastric DLBCL has moved mainly toward organ preservation, while surgery is superior for treatment of intestinal non-Hodgkin lymphoma^19^. In addition, related literature reported that the common pathological types of patients with primary gastric lymphoma included mucosa-associated lymphoid tissue, while the ones of patients with PINHL were not the same as gastric lymphoma^20^. The pathogenic site and pathological type were risk factors that affected the survival of primary gastrointestinal non-Hodgkin lymphoma patients. Intestinal lymphoma patients might be benefited from surgery while chemotherapy could be given the first priority for patients with primary gastric lymphoma^17^. Thus, we separated the lesion sites of the stomach and intestinal tract, included more histological subtypes, and expanded the sample size to carry out the study of PINHL to determine the optimal management. Incidents like occlusion, bleeding, or perforation are common in intestinal lymphoma because of the characteristics of the intestines. In contrast to other published series of intestinal lymphoma, our study mainly focused on the patients who adopted chemotherapy to evaluate the benefit of surgical treatment, in addition to studying the clinical characteristics and management of PINHL alone.

In this population-based cohort study from the SEER database (1983–2015), we reported for the first time that chemotherapy plus surgery treatment in PINHL patients older than 18 years led to better OS and CSS. Considering the inherent biases of a retrospective study, we performed a PSM analysis and demonstrated the stability of the improvement in OS and CSS. At present, surgery for primary intestinal lymphoma has been a debated topic, and there have been scarce evidence-based data. Optimal treatment for primary intestinal lymphoma remains uncertain and controversial. However, current clinical trials tend to admit the benefit of surgery combined with chemotherapy in the treatment of primary intestinal lymphomas, which is in agreement with our conclusion that surgical treatment combined with chemotherapy benefited more on PINHL patients. In a cohort study with 345 patients, Kim et al.^21^ compared the prognosis of intestinal DLBCL between patients treated with surgical resection followed by chemotherapy and those with chemotherapy alone. The 3-year OS rate of the surgery plus chemotherapy group (91%) was higher than that of the chemotherapy alone group (62%), and they concluded that surgery plus chemotherapy was an independent prognostic factor of OS. Lee et al.^22^ studied 76 patients diagnosed with DLBCL of the intestine; they reported a three-year progression-free survival rates (PFS) of 92.2% in the surgery followed by the R-CHOP group compared with 74.8% in the R-CHOP alone group (p = 0.009), while OS was 94.2% and 80.7% (p = 0.049), respectively. Compared with patients treated with R-CHOP alone, those who underwent surgery followed by R-CHOP showed a higher survival rate. In Chinese primary intestinal DLBCL population, R-CHOP immunochemotherapy plus surgery showed a superior prognosis compared with R-CHOP alone and it revealed that radical resection or partial resection combined with immunochemotherapy had no significantly difference^23^.

Similar to other studies^1,5,7,15^, we showed that tumor sites in the small bowel predominated (n = 2024, 57.22%) in the PINHL, and DLBCL was the most common histological subtype (n = 1873, 52.95%) in our study. In particular, we found that the beneficial effect of surgery was significant in patients with DLBCL which was similar with another research^24^. The possible explanation could be that perforation was frequent. Namely, it has been reported that the small intestine is the most common site of perforation, while DLBCL is the most common lymphoma associated with perforation. Moreover, the risk of perforation in aggressive B-cell lymphomas (HR 6.31; p < 0.0001) is higher than in indolent B-cell lymphomas^25^. Hence, surgical intervention is the most effective treatment for such a localized disease with complications. In addition, a study with 581 patients undergoing emergent and elective surgery confirmed a five-year survival benefit with surgery^7^. Since the patients included in our study all underwent chemotherapy and perforation usually occurred after the initiation of chemotherapy, surgical treatment likely contributed a lot to survival. In addition, Roy et al.^26^ thought primary colonic lymphoma patients often presented with advanced disease requiring surgical intervention because of its non-specific symptoms. Therefore, surgery followed by chemotherapy could offer the best prognosis^18^. Present studies mostly were retrospective analyses. However, we considered the role of surgery should be precisely defined by prospective randomized trials. As far as the research on PINHL was concerned, although surgery appeared to be an important part of the treatment algorithm in PINHL, definitive statements about its survival benefit remained controversial due to lack of patient stratification based on timing and indication for surgery^5^.

Primary intestinal lymphoma is primarily diagnosed and staged during exploratory laparotomy with surgical resection. Endoscopic biopsy with computed tomography also plays an important role in diagnosis, but its inherent limitation makes the surgical methods more advantageous. Further selection of patients for treatment was exactly based on the stage of intestinal lymphoma at presentation, followed by the patient’s overall health^6^. Among the histological subtypes of PINHL patients included in our study, the stratification analysis showed that DLBCL showed a better outcome with surgical treatment. Kim et al. also emphasized that surgical resection improved prognosis in patients with localized intestinal DLBCL. In terms of the side effects (fatigue, constipation, diarrhea, insomnia, and dyspnea), the surgery plus chemotherapy treatment still showed a substantially favorable outcome in patients with intestinal DLBCL because the side effects were significantly reduced compared with chemotherapy alone. Although DLBCL might be cured by chemotherapy currently, the benefit from the selection of surgery plus chemotherapy should not be neglected^21^.

As for intestinal lymphoma, difficulties in preoperative pathological diagnosis, unpredicted risk of life-threatening complications such as occlusion, bleeding, or perforation, and rapid tumor necrosis secondary to chemo-/radiotherapy are the main indications for surgical treatment. Preventive surgical resection is sometimes advocated in bulky and localized tumors. A recent study has revealed that surgical resection before chemotherapy may become an effective treatment modality for primary small intestinal NHL^19,22^. Thus, special attention should be paid to the role of preventive surgical treatment in intestinal lymphoma. Although the meta-analysis by Cirocchi et al. confirmed the primary role of chemotherapy in the treatment of primary gastrointestinal lymphoma and showed higher mortality in the surgical group, it pointed out that surgery should be restricted to very selected indications, and the utility of preventive surgery cannot be ignored^27^. Considering the quality of life after surgery, Kim et al.^21^ showed that surgery-associated deterioration of quality of life is acceptable because the benefits of surgery plus chemotherapy outweigh the negative effects. Therefore, scientific management of PINHL may require multimodal treatment by a multidisciplinary team including surgeons, radiologists, hematologists, and gastroenterologists to comprehensively evaluate the curative effect of patients in clinical practices.

The strengths of our retrospective cohort study were as follows: a large number of patients (n = 3537) included in the analysis; application to adjust the model to observe the outcome indicators; use of PSM analysis to limit selection bias. Of note, our study population included patients who had undergone chemotherapy, and we only targeted intestinal lymphoma, which distinguished our study from previous studies. Our study has significant clinical implications for reassessing the prognostic value of surgery for primary intestinal non-Hodgkin lymphoma.

### Limitations

Several limitations existed in our study, mainly inherent to its retrospective nature. Some kind of subjective and objective reasons may have influenced the decision for surgery, which may have biased the results. However, further information about surgery were absent in our study. Moreover, the SEER database is incomplete in that it does not record data about surgical details and treatment-related complications. After all, it is notable that complications are associated with the operation. Although we included patients who underwent chemotherapy, the data about specific chemotherapy regimens were unavailable. Thus, we could not evaluate the effects of combined surgical and specific medical treatments on survival since targeted therapy has been widely used^28^. In the future, more clinical trials and studies about the scientific management of surgical treatment of primary intestinal lymphoma are needed.

Currently, diagnosis and treatment of PINHL are not well characterized and remain controversial. In our large, multicentric, and retrospective cohort study, surgical treatment combined with chemotherapy improved survival in patients with PINHL older than 18 years even after PSM analysis. Surgery plus chemotherapy might be recommended as a beneficial therapeutic strategy for intestinal lymphomas, especially for DLBCL. Further details of surgery included in the studies of primary intestinal lymphomas should be conducted to provide more evidence for optimal treatment algorithms.

## Methods

### Ethics statement

In order to acquire relevant data from the database, we signed the SEER Research Data Agreement (No. 17496-Nov2019) and further searched for data according to the approved guidelines. The extracted data were publicly available and de-identified, and the data analysis was considered as non-human subjects by Office for Human Research Protection, therefore, no approval was required from the institutional review board.

### Database introduction

A retrospective cohort of patients diagnosed with PINHL from January 1983 to December 2015 was extracted from the SEER database. The SEER database collects and publishes cancer incidence and survival conditions from population-based cancer registries covering approximately 28% of the US population at present. A series of 3537 patients were eligible using the National Cancer Institute’s SEER*Stat software (Version 8.3.8) (www.seer.cancer.gov/seerstat) updated in November 2018.

### Inclusion and exclusion criteria

Patients were extracted from the SEER database for the period between 1983 and 2015, and non-Hodgkin lymphoma subtype code was selected based on the World Health Organization classification. The tumor had only one primary field and the data of survival months were complete, available and more than 0 days of survival. Considering the differences between gastric and intestinal lymphoma, we focused exclusively on intestinal non-Hodgkin lymphoma. The primary intestinal area distinguished by the third edition of the International Classification of Diseases for Oncology (ICD-O-3) histology codes (C17.0–C21.8) was defined as the area from the duodenum to the anus, and it involved the small intestine, large intestine, and anus. The exclusion criteria were as follows: patients whose type of reporting source was autopsy only and death certificate only; diagnostic confirmation, the treatment mode of surgery, and Ann Arbor stage were unknown; patients aged < 18 years; patients who had 0–1 month survival; cause-specific death data were missing or unknown. Moreover, only patients treated with chemotherapy were selected for further analysis to improve the accuracy and reliability of survival analyses. Finally, the data of the type of follow-up were available. Figure 1 shows the detailed screening procedure.

### Data collection

Variable selection was based on clinical experience and previous studies examining the risk factors for poor survival. We collected patients’ demographic characteristics, tumor characteristics, treatment data, and survival data. The variables also included gender (females and males), race (White, Black, and other), and marital status (married, non-married, or unknown). Non-married patients included single, unmarried, separated, divorced, widowed, and domestic partners. The year of diagnosis was divided by decade^29^. The reason why we chose patients diagnosed from 1983 to 2015 was that they had complete Ann Arbor staging and surgical information^30^. Staging was defined according to Ann Arbor Stage of lymphoma. Histological subtypes were mainly grouped by common types, such as diffuse large B-cell, follicular, mantle cell, Burkitt, T cell, and others^29^. Based on previous articles, tumor sites specified encompassed the small bowel, ileocecum, colon, and other^1,5,21,22^. Treatment types were recorded as two binary variables, i.e., acceptance of radiation (yes/no) and surgery (yes/no). Surgical treatment was the core of our study, so we divided all the subjects into two groups (non-surgery group and surgery group) to probe the relationship between surgical intervention and survival outcomes.

### Study endpoints

Overall survival (OS) was considered as the primary outcome, and cancer-specific survival (CSS) was defined as the secondary outcome. As a frequently-used and reliable indicator of prognosis, OS was measured from the first date of diagnosis to the date of death of any cause, comprised patients censored at the last follow-up date. CSS, a specific indicator, was defined from the first date of diagnosis until death due to lymphoma or treatment-related causes.

### Statistical analysis

Statistical differences in patients’ demographic characteristics and tumor characteristics between the non-surgery and surgery groups were evaluated using the χ^2^ test for categorical variables and *t* test for continuous variables, respectively. Age was accessed as a continuous variable, while other variables were categorical. We documented continuous and categorical data as mean with standard deviation (normal distribution) and numbers and percentages of the surgery groups (categorical data). The prognostic effect of the various variables on survival was evaluated using univariate analysis, and the covariant screening was performed for each potential confounding variable. Multivariate analyses were performed using the Cox proportional-hazards model, and all confounding factors were adjusted to identify the effect of surgery on long-term survival. The model included covariates as potential confounders in the covariant screening if they changed the estimates of the effect of surgery on survival conditions by more than 10% or were significantly associated with survival. In accordance with the Strengthening the Reporting of observational studies in Epidemiology (STROBE) guidelines, we also showed the unadjusted, slightly adjusted, and fully adjusted equations. Stratified analyses by age, year of diagnosis, Ann Arbor stage, histology, tumor site, and radiation were conducted by using stratified Cox models. Tests for effect modification by subgroup were based on interaction terms between subgroup indicators and surgery, followed by the likelihood ratio test. The subgroup and interaction analyses were adjusted for the confounding factors. Cumulative survival rates and the CSS hazard curve were constructed according to the Kaplan–Meier method and compared using a log-rank test based on adjustments for potential confounding factors.

### Propensity score matching analysis

To further control the confusion, prevent bias, and ensure the credibility of the data, we subsequently performed a propensity score matching (PSM) analysis. The propensity score was a conditional probability of having a designated exposure (non-surgery and surgery) given a set of baseline measured covariates. The propensity score was assessed by using a non-parsimonious multivariate logistic regression model. Considering that the pretreatment variables including age, gender, race, marital status, year of diagnosis, Ann Arbor stage, histology, tumor site, and radiation had a significant independent effect on survival, we decided to add the above variables into the propensity score model.

We matched the non-surgery group and surgery group based on a range of 0.05 of the propensity score, and the matching was implemented with a 1:1 matching protocol without replacement (greedy-matching algorithm)^31^. We chose the matching range of 0.05 because it was commonly used, provided the reasonable balance of the included covariates, and did not lose many treated individuals as unmatchable. To match participants, we performed an automated matching procedure in the EmpowerStats software, which randomly chose a treated individual and an untreated individual (comparator) from the pool of latent comparators to decide whether they conformed to the matching criterion. If the selected comparator was eligible, he or she was matched to the treated individual, and the pair was removed. This procedure was repeated until all treated patients were matched or until no further comparators conformed to the matching criteria^32^. At last, standardized differences of the above covariates < 10% indicated a relatively small imbalance. The above analysis adopted a recommended guideline amended from the STROBE statement^33^.

After matching, paired-data comparisons were assessed using McNemar’s test for binary variables and Student’s *t* test for continuous variables. This matching method has been shown to effectively correct bias from the measured confounders, and further analyses also addressed potential confounders by adjusting for variables. Regression adjustment was applied to remove post-PSM residual confounding bias where it included the covariates with a standardized difference greater than 10%. A series of analyses including univariate and multivariate analysis, stratified and interaction analyses, and the Kaplan–Meier method were operated in the matched cohort to improve inspection efficiency and guarantee the dependability and stability of the results. The comparative risks of primary and secondary outcomes were further adjusted in the matched cohort with the use of a Cox proportional-hazards regression model^31^. The hazard ratios (HRs) with corresponding 95% confidence intervals (CIs) were used to predict the effect of factors on OS and CSS. Two-tailed P values of less than 0.05 were considered as statistically significant. All the analyses were carried out using the statistical software packages R (http://www.R-project.org, The R Foundation) and EmpowerStats (http://www.empowerstats.com, X & Y Solutions, Inc, Boston, MA).

## Supplementary Information

Supplementary Information.
