# Supplementary material for: The impact of surgery on long-term survival of patients with primary intestinal non-Hodgkin lymphomas based on SEER database
Source: Sci Rep. 2021 Nov 29;11:23047. doi: 10.1038/s41598-021-02597-1 (PMC8630038; doi:10.1038/s41598-021-02597-1)
Supplement: Supplementary file 1 — Supplementary Information. [file 41598_2021_2597_MOESM1_ESM.zip › Supplemental figure legend.docx]

**Supplemental figure legend**

**Supplemental Figure** Forest plots for (**A**) overall survival (OS) and (**B**) cancer specific survival (CSS) with surgery plus chemotherapy treatment after PSM (propensity score-matching). Adjusted HR was calculated based on the Cox proportional hazard model. Figure **A** of OS adjusting for age, year of diagnosis, Ann Arbor Stage, histologic and tumor site except the subgroup variable. Figure **B** of CSS adjusting for age, gender, marital status, year of diagnosis, Ann Arbor Stage, histologic, tumor site and radiation except the subgroup variable.
