# Supplementary material for: The impact of surgery on long-term survival of patients with primary intestinal non-Hodgkin lymphomas based on SEER database
Source: Sci Rep. 2021 Nov 29;11:23047. doi: 10.1038/s41598-021-02597-1 (PMC8630038; doi:10.1038/s41598-021-02597-1)
Supplement: Supplementary file 1 — Supplementary Information. [file 41598_2021_2597_MOESM1_ESM.zip › Supplementary Table 1.pdf]

**Supplemental Table1.** Baseline Patient Demographic and Clinical Characteristics after PSM (propensity score-matching).

| | Total(n=2868): n(%) | Non-surgery (n=1434):n(%) | Surgery(n=1434): n(%) | P-value |
|---|---|---|---|---|
| **Age(years)** | 58.32 ± 16.25 | 59.55 ± 15.77 | 57.09 ± 16.64 | <0.0001 |
| **Gender** | | | | 0.055 |
| female | 1001 (34.90%) | 476 (33.19%) | 525 (36.61%) | |
| male | 1867 (65.10%) | 958 (66.81%) | 909 (63.39%) | |
| **Race** | | | | 0.54 |
| White | 2351 (81.97%) | 1165 (81.24%) | 1186 (82.71%) | |
| Black | 185 (6.45%) | 97 (6.76%) | 88 (6.14%) | |
| Other | 320 (11.16%) | 164 (11.44%) | 156 (10.88%) | |
| Unknown | 12 (0.42%) | 8 (0.56%) | 4 (0.28%) | |
| **Marital status** | | | | 0.58 |
| Unmarried | 1104 (38.49%) | 563 (39.26%) | 541 (37.73%) | |
| Married | 1669 (58.19%) | 827 (57.67%) | 842 (58.72%) | |
| Unknown | 95 (3.31%) | 44 (3.07%) | 51 (3.56%) | |
| **Year of diagnosis** | | | | <0.001 |
| 1980s | 125 (4.36%) | 15 (1.05%) | 110 (7.67%) | |
| 1990s | 465 (16.21%) | 155 (10.81%) | 310 (21.62%) | |
| 2000s | 1386 (48.33%) | 712 (49.65%) | 674 (47.00%) | |
| 2010s | 892 (31.10%) | 552 (38.49%) | 340 (23.71%) | |
| **Ann Arbor Stage** | | | | <0.001 |
| I | 995 (34.69%) | 517 (36.05%) | 478 (33.33%) | |
| II | 892 (31.10%) | 368 (25.66%) | 524 (36.54%) | |
| III | 193 (6.73%) | 101 (7.04%) | 92 (6.42%) | |

| | | | | |
|---|---|---|---|---|
| IV | 788 (27.48%) | 448 (31.24%) | 340 (23.71%) | |
| **Histologic** | | | | <0.001 |
| DLBCL | 1468 (51.19%) | 689 (48.05%) | 779 (54.32%) | |
| FL | 358 (12.48%) | 206 (14.37%) | 152 (10.60%) | |
| MCL | 179 (6.24%) | 134 (9.34%) | 45 (3.14%) | |
| BL | 190 (6.62%) | 69 (4.81%) | 121 (8.44%) | |
| TCL | 120 (4.18%) | 45 (3.14%) | 75 (5.23%) | |
| Other | 553 (19.28%) | 291 (20.29%) | 262 (18.27%) | |
| **Tumor site** | | | | <0.001 |
| Small bowel | 1603 (55.89%) | 758 (52.86%) | 845 (58.93%) | |
| Ileocecum | 489 (17.05%) | 178 (12.41%) | 311 (21.69%) | |
| Colon | 549 (19.14%) | 325 (22.66%) | 224 (15.62%) | |
| Other | 227 (7.91%) | 173 (12.06%) | 54 (3.77%) | |
| **Radiation** | | | | 0.003 |
| No | 2615 (91.18%) | 1285 (89.61%) | 1330 (92.75%) | |
| Yes | 253 (8.82%) | 149 (10.39%) | 104 (7.25%) | |

DLBCL: diffuse large B cell; FL: Follicular lymphoma; MCL: Mantle cell lymphoma; BL: Burkitt lymphoma; TCL: T cell lymphoma.
