# Supplementary material for: The impact of surgery on long-term survival of patients with primary intestinal non-Hodgkin lymphomas based on SEER database
Source: Sci Rep. 2021 Nov 29;11:23047. doi: 10.1038/s41598-021-02597-1 (PMC8630038; doi:10.1038/s41598-021-02597-1)
Supplement: Supplementary file 1 — Supplementary Information. [file 41598_2021_2597_MOESM1_ESM.zip › Supplementary Table 2.pdf]

Supplemental Table 2. Univariate analysis of OS and CSS Cox proportion hazard ratio analysis after propensity score matching

**Characteristics**	**OS**		**CSS**	
	**HR (95% CI)**	**P value**	**HR (95% CI)**	**P value**
**Age(years)**	1.03 (1.02, 1.03)	<0.0001	1.01 (1.01, 1.02)	<0.0001
**Gender**				
female	1		1	
male	1.14 (1.01, 1.28)	0.0311	1.28 (1.11, 1.47)	0.0006
**Race**				
White	1		1	
Black	1.23 (1.00, 1.52)	0.0493	1.24 (0.97, 1.58)	0.0851
Other	0.81 (0.68, 0.98)	0.0288	0.89 (0.72, 1.10)	0.2671
Unknown	0.39 (0.10, 1.55)	0.1811	0.52 (0.13, 2.10)	0.3621
**Marital status**				
Unmarried	1		1	
Married	0.80 (0.72, 0.90)	0.0001	0.72 (0.64, 0.83)	<0.0001
Unknown	0.90 (0.66, 1.24)	0.5352	0.84 (0.58, 1.21)	0.3492
**Year of diagnosis**				
1980s	1		1	
1990s	1.03 (0.82, 1.29)	0.788	1.21 (0.92, 1.59)	0.183
2000s	0.52 (0.42, 0.64)	<0.0001	0.54 (0.41, 0.71)	<0.0001
2010s	0.36 (0.28, 0.46)	<0.0001	0.36 (0.27, 0.48)	<0.0001
**Ann Arbor Stage**				
I	1		1	
II	1.01 (0.88, 1.16)	0.9104	1.12 (0.94, 1.33)	0.1914
III	1.04 (0.81, 1.33)	0.7621	1.21 (0.91, 1.60)	0.1912
IV	1.65 (1.44, 1.88)	<0.0001	1.85 (1.57, 2.17)	<0.0001
**Histologic**				
DLBCL	1		1	

FL	0.41 (0.32, 0.51)	<0.0001	0.33 (0.25, 0.45)	<0.0001
MCL	0.84 (0.66, 1.07)	0.1529	0.89 (0.68, 1.17)	0.4104
BL	0.77 (0.60, 0.98)	0.0345	0.82 (0.62, 1.09)	0.1672
TCL	2.19 (1.75, 2.74)	<0.0001	2.56 (2.01, 3.27)	<0.0001
Other	1.11 (0.97, 1.28)	0.1277	1.17 (1.00, 1.38)	0.0557
**Tumor site**				
Small bowel	1		1	
Ileocecum	0.91 (0.78, 1.07)	0.2492	0.82 (0.68, 0.99)	0.0388
Colon	1.21 (1.05, 1.39)	0.0085	1.10 (0.93, 1.31)	0.2509
Other	1.25 (1.02, 1.52)	0.0293	1.35 (1.08, 1.69)	0.0078
**Radiation**				
No	1		1	
Yes	1.06 (0.88, 1.28)	0.5482	1.19 (0.96, 1.47)	0.1051

OS: overall survival; CSS: cancer specific survival; HR: hazard ratio; DLBCL: diffuse large B cell; FL: Follicular lymphoma; MCL: Mantle cell lymphoma; BL: Burkitt lymphoma; TCL: T cell lymphoma.
