# Supplementary figures and images for: The impact of surgery on long-term survival of patients with primary intestinal non-Hodgkin lymphomas based on SEER database
Source: Sci Rep. 2021 Nov 29;11:23047. doi: 10.1038/s41598-021-02597-1 (PMC8630038; doi:10.1038/s41598-021-02597-1)

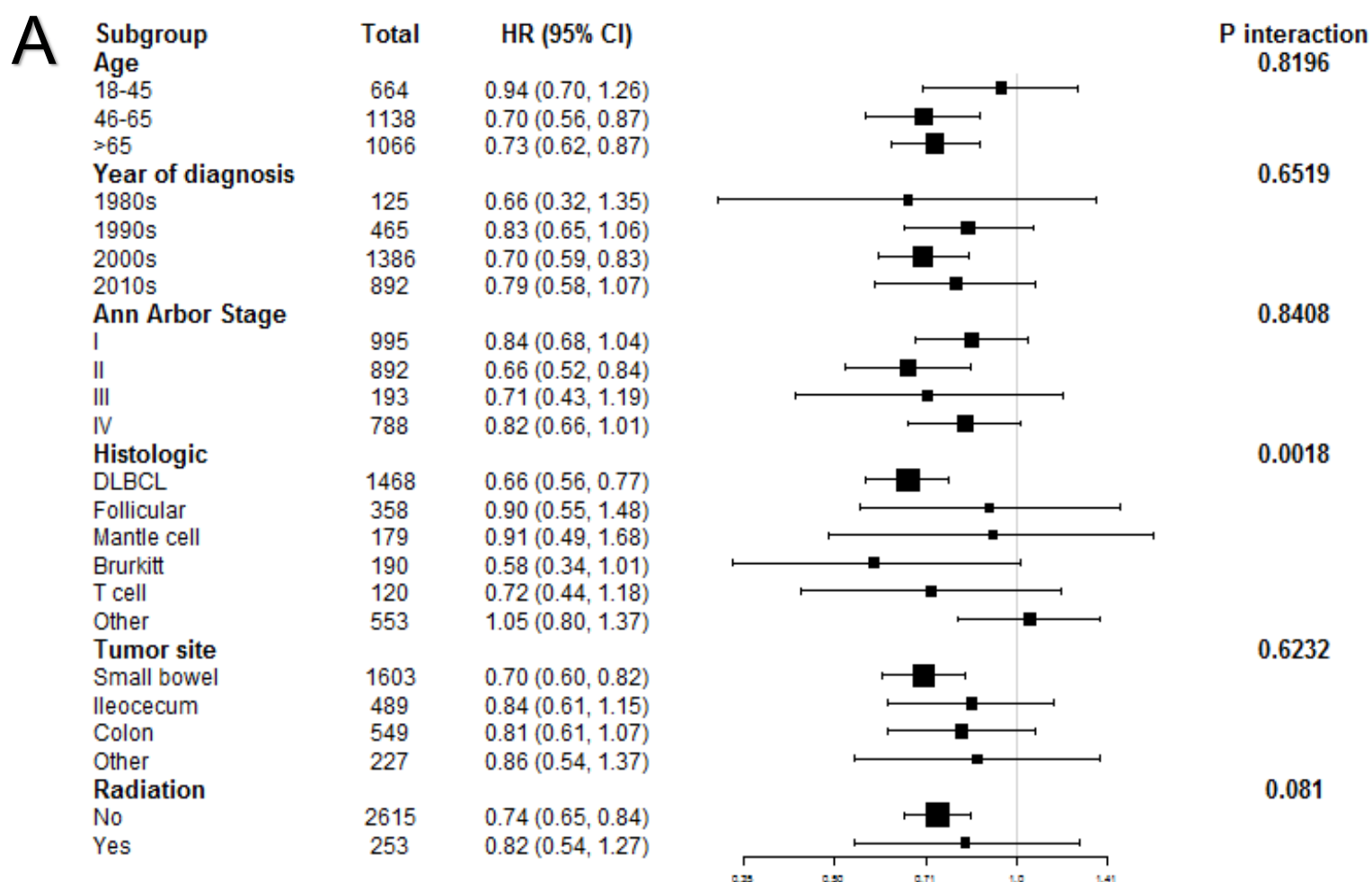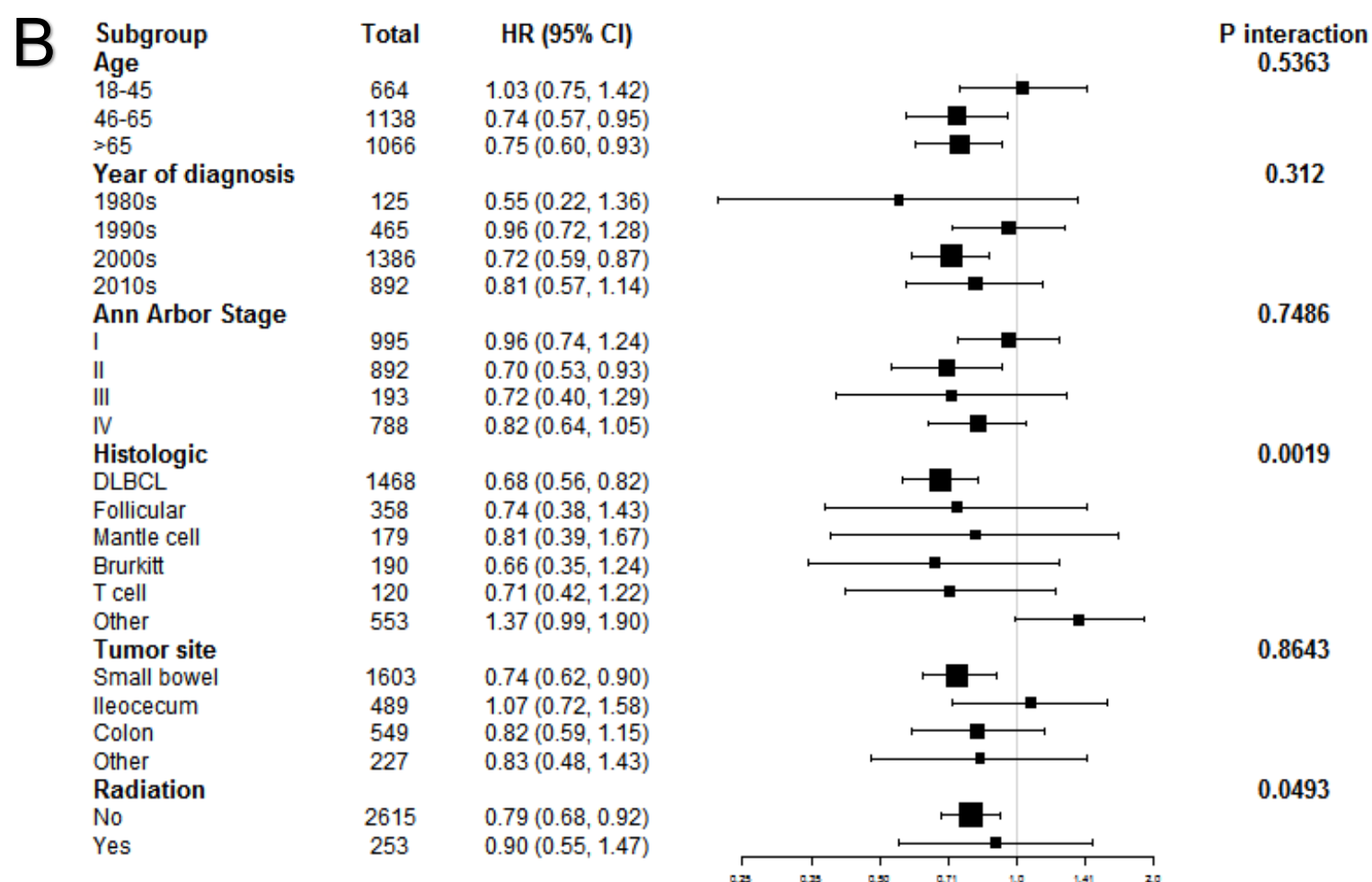

Supplement: Supplementary file 1 — Supplementary Information. [file 41598_2021_2597_MOESM1_ESM.zip › Supplementary Figure 1.pdf]
